# Enhanced Ferroelectric and Piezoelectric Properties of (1−*x*)PMN-*x*PT Ceramics Based on a Partial Oxalate Process

**DOI:** 10.3390/ma11112247

**Published:** 2018-11-12

**Authors:** Jinhwan Kim, Sanghyun Yoon, Jae-Hoon Ji, Young-Ho Ko, Kyung-Ho Cho, Sang-Kwon Lee, Jung-Hyuk Koh

**Affiliations:** 1 School of Electrical and Electronics Engineering, Chung-Ang University, Seoul 06974, Korea; kjh2203kjh@naver.com (J.K.); moon_light90@naver.com (S.Y.); hoon2441@naver.com (J.-H.J.); 2 Agency for Defense Development, Daejeon 34186, Korea; kohjunghyuk@hanmail.net (Y.-H.K.); redskin99@naver.com (K.-H.C.); 3 Department of Physics, Chung-Ang University, Seoul 06974, Korea; sangkwonlee@cau.ac.kr

**Keywords:** piezoelectric, PMN-PT, partial oxalate process

## Abstract

The pyrochlore phase in ferroelectric and piezoelectric materials is the main obstacle device application due to its poor electrical properties. Especially, the pyrochlore phase is frequently observed in the perovskite-based metal-oxide materials including piezoelectric and ferroelectric ceramics, which are based on solid-state reaction methods for fabrication. To overcome these problems, advanced innovative methods such as partial oxalate process will be investigated. In this method, crystalized magnesium niobite (MN) and lead titanate (PT) powders will be coated with a certain amount of lead oxalate and, then, the calcination process can be carried out to form the PMN-PT without pyrochlore phase. In this study, (1−*x*)PMN-*x*PT ceramics near the morphotropic phase boundary (MPB), with compositions of *x* = 0.25–0.40, have been prepared employing the partial oxalate method at various temperatures. The crystalline, microstructure, and piezoelectric properties of (1−*x*)PMN-*x*PT ceramics depending on the sintering temperature were intensively investigated and discussed. By optimizing the sintering temperature and compositions from the PMN-PT ceramics, the maximum value of the piezoelectric charge coefficient (*d_33_*) of 665pC/N, planar electromechanical coupling factor (*k_p_*) of 77.8%, dielectric constant (*ε_r_*) of 3230, and remanent polarization (*P_r_*) of 31.67 μC/cm^2^ were obtained.

## 1. Introduction

Pb(Mg_1/3_Nb_2/3_)O_3_ (PMN) is a well-known relaxor ferroelectric material that has a high dielectric constant (around −10 °C) with broad diffuse and dispersive phase transition [1,2]. PbTiO_3_ (PT) is a normal ferroelectric material, it shows piezoelectric behavior and has a Curie temperature (*T_c_*) of 490 °C [3,4]. By employing a solid solution process, PT composition can be varied in the PMN-PT ceramics. PMN-PT can be employed in varied devices such as sensors, actuators, and transducers to substitute PZT materials because of its outstanding properties [5,6]. Especially, (1−*x*)PMN-*x*PT ceramics in the PT composition range between 0.32 and 0.35 mol, and show a morphotropic phase boundary (MPB) between the rhombohedral and tetragonal phases [7,8,9]; it also shows high piezoelectric and ferroelectric properties. However, the formation of a lead niobate-based pyrochlore phase during the beginning stages of sintering process, which reduces the electrical properties of the material, is a major obstacle observed in the fabrication of PMN-PT ceramics. Therefore, several studies have been performed to eliminate the pyrochlore phase during synthesis. The various properties of PMN-PT ceramics based on different synthesis methods are listed in Table 1 [10,11,12,13,14,15,16,17,18,19,20]. As shown in Table 1, PMN-PT piezoelectric ceramics prepared by the partial oxalate method tend to show a higher piezoelectric charge coefficient (*d_33_*) of 665 pC/N with a higher density of 8.23 g/cm^3^. Among the several processing methods used to fabricate PMN-PT ceramics to form pure pervoskite phase, the Columbite precursor method has been widely used due to higher yield of perovskite phase content [21]. The Columbite precursor method can synthesize raw material corresponding to B-site in perovskite structure in ABO_3_, prior to the reaction with lead. However, this Columbite method has demerits in the formation of pyrochlore phase during the sintering process. Also, several other fabrication methods such as sol-gel method, KCl molten salt method, and hot pressing have been performed and analyzed to improve piezoelectric properties by eliminating the pyrochlore phase. However, most of them leave a residual pyrochlore phase in the sintered ceramics [8,22,23].

The partial oxalate method is an innovative technique to remove the pyrochlore phase of lead-based ceramics. This method has been successfully investigated for the synthesis of PZT, PLZT and PMN [24,25].

In this study, after making B-site compound of ABO_3_ structure using columbite precursor method, MN and PT powders were coated with the needed amount of lead oxalate followed by calcination to form the PMN-PT. (1−*x*)PMN-*x*PT ceramics were prepared using a partial oxalate method to remove the pyrochlore phase at various sintering temperatures. The influence of synthesis method on the dielectric, piezoelectric, and ferroelectric properties of PMN-PT ceramics was investigated. Also, we have investigated (1−*x*)PMN-*x*PT ceramics, focusing on the effects of MPB region according with PT content with respect to using applications. 

## 2. Materials and Methods

(1−*x*)PMN-*x*PT (*x* = 0.250, 0.300, 0.325, 0.350, 0.400 mol) ceramics were prepared using the partial oxalate method [26]. Reagent-grade MgO (Sigma Aldrich, Saint Louis, MI, USA, 99.0%), Nb_2_O_5_ (Sigma Aldrich, 99.9%), TiO_2_ (Sigma Aldrich, 99.9%), Pb(NO_3_)_2_ (Sigma Aldrich, 99.0%), and oxalic acid (Sigma Aldrich, 99.0%) powders were used as starting materials. The weighed powders (MgO, Nb_2_O_5_) according to the stoichiometry of composition were mixed in high-purity ethanol (EMD Millipore, Burlington, MA, USA, 99.9%) for 6 h by planetary ball milling with a zirconia ball, then calcined at 1100 °C for 4 h. The stoichiometric MN and TiO_2_ powders were mixed in high-purity ethanol for 6 h by planetary ball milling with zirconia balls, then calcined at 1100 °C for 4 h. The weight ratio between the balls and powders were 10:1. Until now, very rare papers were reported for the partial oxalate processing techniques for the PMN ceramics case. Also, there is no papers for the PMN-PT piezoelectric ceramics with improved piezoelectric properties by comparing existence of pyrochlore phase. The partial oxalate process was carried out at 90 °C because this temperature enhances the reaction with oxalic acid to form soluble Pb(MNT)OC_2_O_4_·H_2_O species. The processing steps of the oxalic process may be described by the following equations:

(MNT)O_2_ + H_2_C_2_O_4_ + H_2_O → (MNT)OC_2_O_4_ + 2H_2_O (1)

(MNT)OC_2_O_4_ + Pb(NO_3_)_2_ + H_2_C_2_O_4_ + H_2_O → Pb(MNT)OC_2_O_4_·H_2_O_(s)_ + 2HNO_3_ (2)

The coated Pb(MNT)OC_2_O_4_·H_2_O powders were washed several times, first with distilled water and then with high-purity ethanol, prior to being separated by filtration. The oxalate-coated Pb(MNT)OC_2_O_4_·H_2_O powders were dried at 120 °C, then calcined at 750 °C for 4 h. The (1−*x*)PMN-*x*PT powders were crushed and milled using high-purity ethanol as a ball milling for 12 h and calcined again at 750 °C for 4 h. The (1−*x*)PMN-*x*PT powders were pressed into a disc (12 mm diameter × 1.5 mm thickness) at a pressure of 294 MPa using polyvinyl alcohol (PVA) as a binder. After burning off the PVA (600 °C, 1 h) the pellets were sintered at 1200–1300 °C (1200, 1225, 1250, 1275 and 1300 °C) for 4 h. 

The structures were examined by X-ray diffraction (XRD) analysis (New D8-Advance, Bruker-AXS, Billerica, MA, USA). The measurements were carried out from 20–70° by 0.02° differences. The wave-length of incident beam is around 1.54 Å from CuK_α_ radiation source. The microstructures of the ceramics were observed by field emission scanning electron microscopy (FE-SEM) (SIGMA, Carl Zeiss, Upper Cohen, Germany). The frequency-dependent and temperature-dependent dielectric constant (*ε_r_*) of these ceramics were analyzed. Polarization versus electric (P-E) hysteresis loops and strain versus electric (S-E) loops of the ceramic were then measured. The piezoelectric charge coefficient (*d_33_*) and planar electromechanical coupling factor (*k_p_*) were measured by the Berlin-court quasi-static meter (YE2730A, APC International, Ltd., Mackeyville, PA, USA) and impedance analyzer (Agilent 4294A, Agilent Technologies, Santa Clara, CA, USA), respectively. 

## 3. Results and Discussion

**Figure 1:**
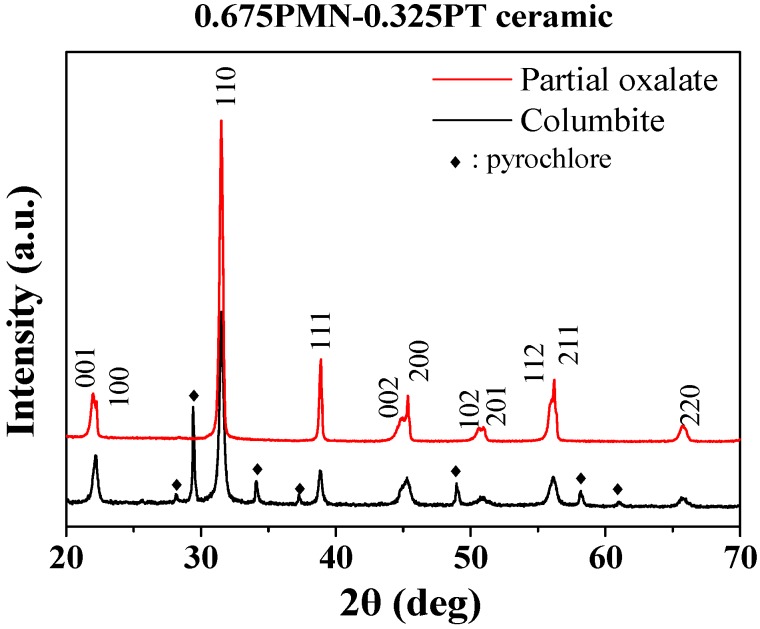
X-ray diffraction *θ*–2*θ* scans with CuK_α_ radiation of the 0.675PMN-0.325PT ceramics synthesized by Columbite method and partial oxalate process sintered at 1275 °C.

Figure 1 presents XRD patterns of 0.675PMN-0.325PT ceramics synthesized by the Columbite method and partial oxalate process sintered at 1275 °C. The PMN-PT ceramic prepared by the partial oxalate process was found to produce a pure perovskite phase, while the PMN-PT ceramic obtained by the Columbite method was found to produce a pyrochlore phase. 

**Figure 2:**
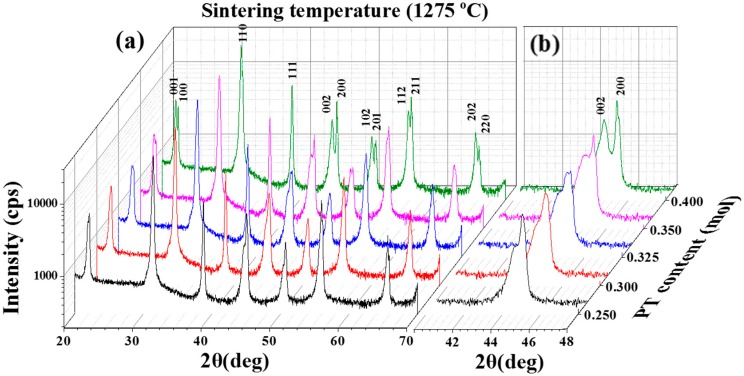
X-ray diffraction *θ*–2*θ* scans with CuK_α_ radiation for the (1−*x*)Pb(Mg_1/3_Nb_2/3_)O_3_-*x*PbTiO_3_ (*x* = 0.250, 0.300, 0.325, 0.350, 0.400 mol) ceramics sintered at 1275 °C (**a**) from 20 to 70 °and (**b**) from 40 to 48 °.

Figure 2a shows XRD patterns using a log-scale of the (1−*x*)PMN-*x*PT (*x* = 0.250, 0.300, 0.325, 0.350, 0.400 mol) ceramics sintered at 1275 °C. The XRD patterns were measured from 20 to 70° at 0.02° intervals for 0.5 h, respectively. Although the XRD figures were shown in the log scale in the Y -axis, any pyrochlore phase was not observed. As shown in the Figure 2a, a pure perovskite phase was observed for all specimens without any pyrochlore phase. This indicates that the partial oxalate method effectively removed the pyrochlore phase and assisted to form the PMN-PT ceramic. Figure 2b describes the Bragg reflection of the (1−*x*)PMN-*x*PT from 44 to 46°. A PT composition of 0.250 mol shows the rhombohedral phase. Then, as the PT composition increased up to 0.400 mol, the (200) peak positions moved to the higher angle, and confirmed (002)/(200) peak splitting at PT composition of 0.400 mol. This indicated that mixed rhombohedral and tetragonal phases (MPB region) are present between the PT composition from 0.325 to 0.350 mol. Also, that composition could show the highest piezoelectric, dielectric, and ferroelectric properties compared with other specimens. 

**Figure 3:**
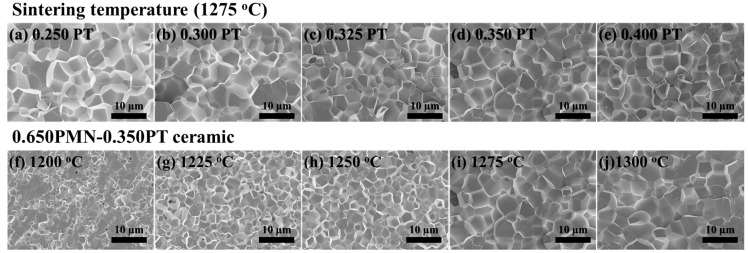
FE-SEM micrographs of the cross-sections after thermal etching; (**a**) 0.750Pb(Mg_1/3_Nb_2/3_)O_3_-0.250PbTiO_3_; (**b**) 0.700Pb(Mg_1/3_Nb_2/3_)O_3_-0.300PbTiO_3;_ (**c**) 0.675Pb(Mg_1/3_Nb_2/3_)O_3_-0.325PbTiO_3_, (**d**) 0.650Pb(Mg_1/3_Nb_2/3_)O_3_-0.350PbTiO_3_, and (**e**) 0.600Pb(Mg_1/3_Nb_2/3_)O_3_-0.400PbTiO_3_, ceramics sintered at 1275 °C and the 0.650Pb(Mg_1/3_Nb_2/3_)O_3_-0.350PbTiO_3_ ceramics sintered at (**f**) 1200 °C, (**g**) 1225 °C, (**h**) 1250 °C, (**i**) 1275 °C, and (**j**) 1300 °C.

Figure 3 displays cross-sectional FE-SEM images of the (1−*x*)PMN-*x*PT (*x* = 0.250, 0.300, 0.325, 0.350, 0.400 mol) ceramics sintered at 1275 °C and the 0.650PMN-0.350PT ceramics depending on a sintering temperature from 1200 to 1300 °C. After polishing cross-sections of all specimens, they were thermally etched for 10 min at a temperature of 100 °C lower than the sintering temperature.

Platinum (Pt) coating using an ion-coater was carried out for 120 s, and all samples were measured under the same conditions at 7000 magnifications. 0.750PMN-0.250PT ceramics showed a relatively large grain size of approximately 6.75 μm compared with that of other composition ceramics. The increasing amount of PT substitution in the PMN composition can cause not only lattice distortion but also dwindle the grain size of these ceramics, as shown in the Figure 3a. Otherwise, when the sintering temperature increased up to 1300 °C, grain size was increased compared with 0.650PMN-0.350PT sintered at 1200 °C as shown in the Figure 3b. As increasing the sintering temperature, grain size increases because of stress relaxation. This result could be explained by the phenomenological kinetic grain growth equation [27,28].

**Figure 4:**
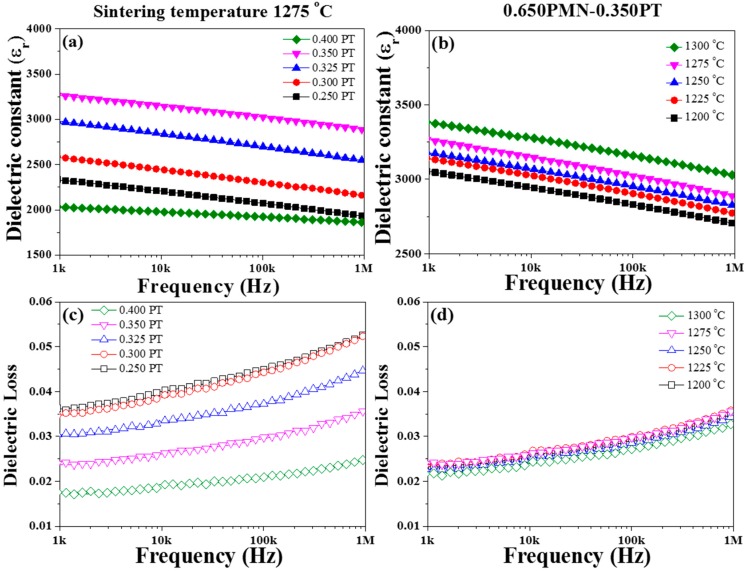
Frequency-dependent dielectric constant and dielectric losses; (**a**) dielectric constant and (**c**) dielectric losses the (1−*x*)Pb(Mg_1/3_Nb_2/3_)O_3_-*x*PbTiO_3_ (*x* = 0.250, 0.300, 0.325, 0.350, 0.400 mol) ceramics sintered at 1275 °C and (**b**) dielectric constant and (**d**) dielectric losses the 0.650Pb(Mg_1/3_Nb_2/3_)O_3_-0.350PbTiO_3_ ceramics sintered from 1200 to 1300 °C.

Figure 4 illustrates the dielectric constants (*ε_r_*) and dielectric losses of the (1−*x*)PMN-*x*PT (*x* = 0.250, 0.300, 0.325, 0.350, 0.400 mol) ceramics sintered at 1275 °C and the 0.650PMN-0.350PT ceramics sintered from 1200 to 1300 °C depending on the frequency range. The dielectric properties were measured from 1 kHz to 1 MHz. The *ε_r_* was calculated from the computed capacitance by an impedance analyzer through the following equation:

(3) $$\;\varepsilon_{r}=\;\frac{C\cdot d}{\varepsilon_{0}\cdot A}\;$$

where *C* was the capacitance of the sample, *d* was the sample thickness, *A* was the area of the electrode, and *ε*_0_ was the dielectric constant of a vacuum (8.854 × 10^−14^ F/cm). Based on the equation, the *ε_r_* of PT composition of 0.350 mol is higher than that of other compositions, with a maximum value of 3230 at 1 kHz. Then, it decreases when PT composition is above 0.350 mol. This tendency is attributed to the occurrence of phase transition that was previously verified by analysis of XRD pattern as shown in Figure 2b. 

The dielectric losses of the (1−*x*)PMN-*x*PT (*x* = 0.250, 0.300, 0.325, 0.350, 0.400 mol) ceramics sintered at 1275 °C were decreased as increasing the PT composition, indicating that PT can usefully decrease dielectric loss. Additionally, we found that by increasing the sintering temperature up to 1300 °C, the *ε_r_* value was increased by about 1.07 times at 1 kHz compared to that of 1200 °C as shown in the Figure 4b. The measured *ε_r_* of 0.650PMN-0.350PT ceramic sintered at 1300 °C is around 3278 at 1 kHz. This result correlates to grain size and sintering temperature. As increasing the sintering temperature, grain size can cause not only a decrease of grain-boundary volume but also increase in the *ε_r_* of 0.650PMN-0.350PT ceramic. 

**Figure 5:**
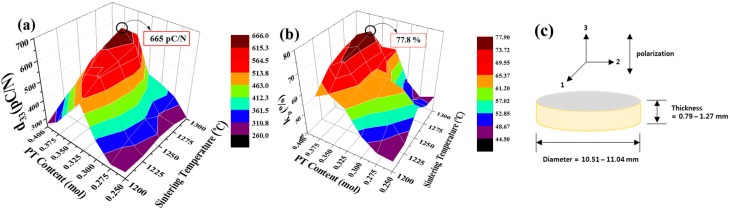
(**a**) piezoelectric charge coefficient (*d_33_*), (**b**) planar electromechanical coupling factor (*k_p_*), and (**c**) the graphic geometry used for the (1−*x*)Pb(Mg_1/3_Nb_2/3_)O_3_-*x*PbTiO_3_ (*x* = 0.250, 0.300, 0.325, 0.350, 0.400 mol) ceramics sintered from 1200 to 1300 °C.

Figure 5a shows a piezoelectric charge coefficient (*d_33_*) of the (1−*x*)PMN-*x*PT (*x* = 0.250, 0.300, 0.325, 0.350, 0.400 mol) ceramics sintered from 1200 to 1300 °C. The *d_33_* increased with increasing PT composition up to 0.350 mol and sintering temperature up to 1275 °C, with a maximum *d_33_* of 665 pC/N. Figure 5b displays planar electromechanical coupling factor (*k_p_*) of the (1−*x*)PMN-*x*PT (*x* = 0.250, 0.300, 0.325, 0.350, 0.400 mol) ceramics sintered from 1200 to 1300 °C. These tendencies of *k_p_* were like those of *d_33_*. The *k_p_* were calculated by using the following equation [29]:

(4) $$\;k_{p}=\;\sqrt{2.529\;\times \;\frac{\left(f_{a}^{2}-\;f_{r}^{2}\right)}{f_{r}^{2}}}\;$$

where *f_a_* and *f_r_* are the anti-resonance and the resonance frequency, respectively. The maximum value of *k_p_* was 77.8% in 0.650PMN-0.350PT at 1275 °C. As a result, these increased *d_33_* and *k_p_* were related with each composition and MPB region, as discussed in Figure 2b.

**Figure 6:**
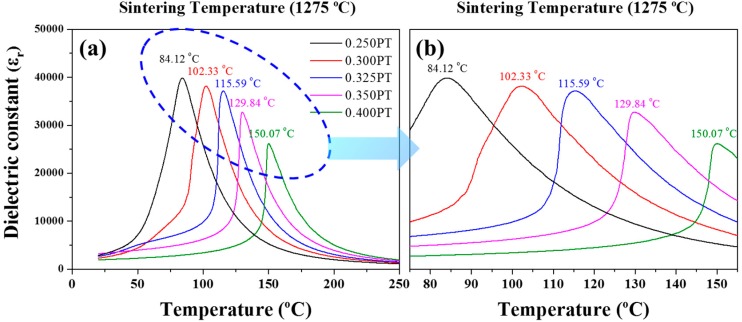
Temperature-dependent dielectric constant (*ε_r_*) at 1 kHz for the (1−*x*)Pb(Mg_1/3_Nb_2/3_)O_3_-*x*PbTiO_3_ (*x* = 0.250, 0.300, 0.325, 0.350, 0.400 mol) ceramics sintered at 1275 °C. (**a**) Dielectric constant (*ε_r_*) from 25 °C to 250 °C; (**b**) dielectric constant (*ε_r_*) from 75 °C to 155 °C.

Figure 6 illustrates the temperature-dependent dielectric constant (*ε_r_*) at 1 kHz for the (1−*x*)PMN-*x*PT (*x* = 0.250, 0.300, 0.325, 0.350, 0.400 mol) ceramics sintered at 1275 °C. The (1−*x*)PMN-*x*PT ceramic samples showed a temperature of maximum dielectric constant (*T_m_*) of 84.12, 102.33, 115.59, 129.84, and 150.07 °C for PT composition of 0.250, 0.300, 0.325, 0.350, 0.400 mol, respectively. As shown in Figure 6, the *T_m_* increased with increasing contents of PT up to 0.400 mol, reaching a maximum value of 150.07 °C, because PT has a Curie temperature of 490 °C. Also, PMN-PT ceramics show ferroelectric properties at temperatures above *T_m_* unlike normal ferroelectric materials because of relaxor property. 

**Figure 7:**
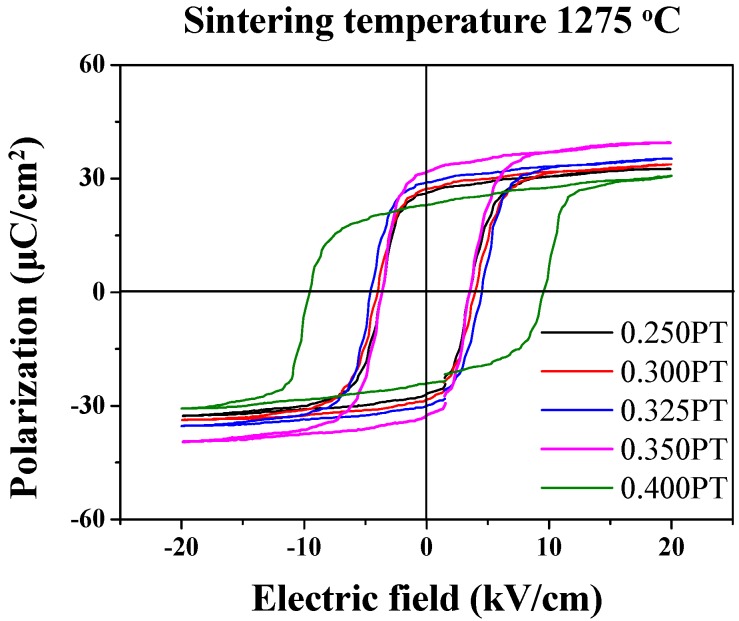
P-E hysteresis loop for the (1−*x*)Pb(Mg_1/3_Nb_2/3_)O_3_-*x*PbTiO_3_ (*x* = 0.250, 0.300, 0.325, 0.350, 0.400 mol) ceramics sintered at 1275 °C.

Figure 7 shows P-E hysteresis loop for the (1−*x*)PMN-*x*PT (*x* = 0.250, 0.300, 0.325, 0.350, 0.400 mol) ceramics sintered at 1275 °C. The P-E hysteresis loops were measured by a Sawyer–Tower circuit under an applied electric field of 20 kV/cm at 0.1 Hz. With an increasing PT composition, the coercive electric field (*E_c_*) was decreased. Whereas saturation polarization (*P_s_*) and remnant polarization (*P_r_*) increased up to 39.42 μC/cm^2^ and 31.67 μC/cm^2^, respectively, and the P-E loop revealed the presence of a typical hysteresis loop of ferroelectric materials. Also, 0.600PMN-0.400PT ceramics showed large *E_c_* which is attributed to smaller grain size (Figure 3). It is well known that as grain size decrease, grain boundary area increases and most of the field is lost. Additionally, because of built-in bias fields, accumulation of space charge and unbalances of electrodes, the P-E loops show discontinuity [30,31].

**Figure 8:**
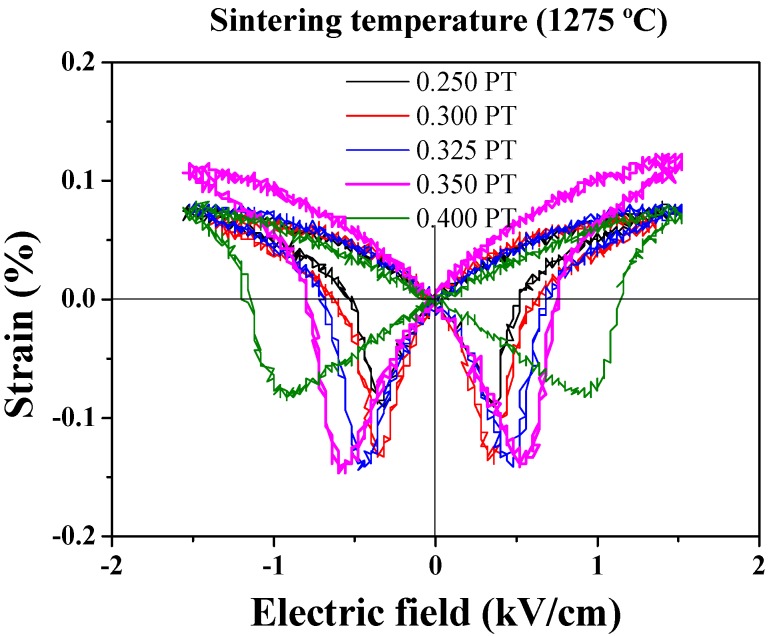
Bipolar S-E loop for the (1−*x*)Pb(Mg_1/3_Nb_2/3_)O_3_-*x*PbTiO_3_ (*x* = 0.250, 0.300, 0.325, 0.350, 400 mol) ceramics sintered at 1275 °C.

Figure 8 shows bipolar S-E loop for the (1−*x*)PMN-*x*PT (*x* = 0.250, 0.300, 0.325, 0.350, 400 mol) ceramics sintered at 1275 °C. The strain increased up to a PT composition of 0.350 mol and the maximum value of strain was 0.12%. However, upon further increasing the PT composition, the strain began to decrease. Negative strain behavior of all specimens was observed. This result agreed well with the previous data for *ε_r_*, *d_33_*, *k_p_* of (1−*x*)PMN-*x*PT ceramics sintered at 1275 °C.

**Figure 9:**
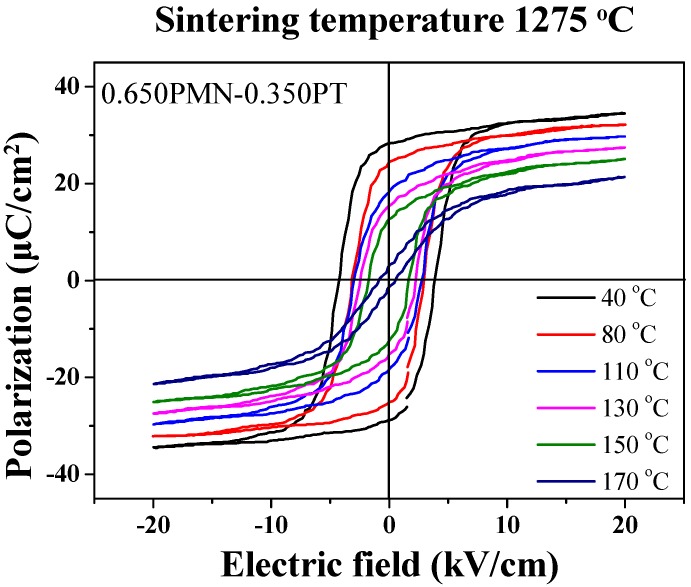
Temperature-dependent P-E hysteresis loop for the 0.650Pb(Mg_1/3_Nb_2/3_)O_3_-0.350PbTiO_3_ ceramic sintered at 1275 °C.

Figure 9 shows a P-E hysteresis loop for the 0.650PMN-0.350PT ceramic from 40 to 170 °C. In all P-E loop data, the observed *P_r_* and *E_c_* decreased as the temperature increased and showed a ferroelectric property at temperatures above *T_m_*, as discussed in Figure 6. The P-E hysteresis loop measured from 40 to 150 °C showed typical ferroelectric behavior. However, the P-E hysteresis loops changed at 170 °C, exhibiting paraelectric properties, meaning that an increased temperature caused phase transition from ferroelectric to paraelectric phase.

## 4. Conclusions

The (1−*x*)PMN-*x*PT ceramics with outstanding piezoelectric and ferroelectric properties have been investigated and analyzed based on a partial oxalate process. As mentioned in XRD patterns, all specimens showed pure pervoskite structure without the pyrochlore phase. At the 0.325 and 0.350 mol of PT near the MPB region between the rhombohedral-tetragonal phase, higher piezoelectric properties were observed. We observed the maximum value of the *d_33_* of 665pC/N and *k_p_* of 77.8% from the 0.650PMN-0.350PT ceramic sintered at 1275 °C. The relaxor ferroelectric property of (1−*x*)PMN-*x*PT ceramics was confirmed through temperature-dependent *ε_r_* and P-E loop. Therefore, these outstanding properties suggest that 0.650PMN-0.350PT ceramics sintered at 1275 °C could be suitable for various applications substitutes for PZT as the relaxor ferroelectric material.

## Figures and Tables

**Table 1:** Comparison of various properties of (1−*x*)PMN-*x*PT ceramics based on different synthesis methods.

Method	*d_33_* (pC/N)	*k_p_* (%)	*ρ* (g/cm^3^)	*ε_r_* (1 kHz)	*T_c_* (°C)	Ref.
Mixed oxide	430	56.4	7.85	2547	162	[10]
Mechanochemical	570	-	-	-	171	[11]
Columbite	610	-	-	6350	-	[12]
Columbite	499	52	-	4830	180	[13]
Columbite	530	-	7.8	-	153	[14]
Spark plasma sintering	350	57	-	-	-	[15]
Spark plasma sintering	590	63.4	-	-	160	[16]
Molten salts	660	-	-	-	165	[17]
Molten salts	540	66	7.73	-	144.5	[18]
Partial oxalate	590	57	7.828	2617.9 (100 Hz)		[19]
Partial oxalate	581	56.5	7.97	-	151	[20]
Partial oxalate	665	77.8	8.23	3230	129.84	Our sample
